# Nitrogen acquisition by plants and microorganisms in a temperate grassland

**DOI:** 10.1038/srep22642

**Published:** 2016-03-10

**Authors:** Qianyuan Liu, Na Qiao, Xingliang Xu, Xiaoping Xin, Jessie Yc Han, Yuqiang Tian, Hua Ouyang, Yakov Kuzyakov

**Affiliations:** 1 Key Laboratory of Ecosystem Network Observation and Modeling, Institute of Geographic Sciences and Natural Resources, Chinese Academy of Sciences, Beijing 100101, China; 2 University of Chinese Academy of Sciences, Beijing 100049, China; 3 Key Laboratory of Tropical Forest Ecology, Chinese Academy of Sciences, Xishuangbanna Tropical Botanical Garden, Menglun, Mengla, Yunnan 666303, China; 4 Hulunber Grassland Ecosystem Observation and Research Station, Institute of Agricultural Resources and Regional Planning of Chinese Academy of Agricultural Sciences, Beijing, China 100081; 5 The Center for Integrative Conservation, Xishuangbanna Tropical Botanical Garden, Menglun, Chinese Academy of Sciences, Mengla, Yunnan 666303, China; 6 State Key Laboratory of Earth Surface Processes and Resource Ecology, Beijing Normal University, No. 19, XinJieKouWai Street, Haidian District, Beijing 100875, China; 7 Department of Soil Science of Temperate Ecosystems and Department of Agricultural Soil Science, University of Göttingen, Göttingen, Germany; 8 Institute of Environmental Sciences, Kazan Federal University, Kazan, Russia

## Abstract

Nitrogen (N) limitation is common in most terrestrial ecosystems, often leading to strong competition between microorganisms and plants. The mechanisms of niche differentiation to reduce this competition remain unclear. Short-term ^15^N experiments with NH_4_^+^, NO_3_^−^, and glycine were conducted in July, August and September in a temperate grassland to evaluate the chemical, spatial and temporal niche differentiation by competition between plants and microorganisms for N. Microorganisms preferred NH_4_^+^ and NO_3_^−^, while plants preferred NO_3_^−^. Both plants and microorganisms acquired more N in August and September than in July. The soil depth had no significant effects on microbial uptake, but significantly affected plant N uptake. Plants acquired 67% of their N from the 0–5 cm soil layer and 33% from the 5–15 cm layer. The amount of N taken up by microorganisms was at least seven times than plants. Although microorganisms efficiently compete for N with plants, the competition is alleviated through chemical partitioning mainly in deeper soil layer. In the upper soil layer, neither chemical nor temporal niche separation is realized leading to strong competition between plants and microorganisms that modifies N dynamics in grasslands.

Plants and microorganisms compete for the same soil resources, but they are mutually dependent on each other^1^. Soil microorganisms need labile organic substances from plants in the form of litter and root exudates^2^,^3^,^4^ to mineralize nutrients from organic to inorganic forms. Plants rely on nutrient supplies mediated by soil microorganisms^4^,^5^,^6^. Plant productivity and soil microbial activities are often tightly coupled, especially in nutrient-poor ecosystems^2^,^6^. Understanding how plants and microorganisms acquire limited nutrients from soils is essential for understanding carbon (C) and nitrogen (N) cycles.

Nitrogen is a fundamental nutrient for plant growth and metabolism but limited in most terrestrial ecosystems^7^, causing strong competition for available N between roots and soil microorganisms^8^,^9^. Studies have explored plant-microbial competition for N to understand the mechanisms responsible for plant productivity^6^, species coexistence^10^,^11^, and ecological consequences of this competition in various terrestrial ecosystems. The consequences of competition often lead to: i) limitation on plant growth, ii) reduced microbial mineralization, and iii) increased competition for N between coexisting plant species.

The old paradigm for terrestrial N cycling assumed that plants were only capable of using inorganic N (i.e., NH_4_^+^ and NO_3_^−^), mineralized by microorganisms from organic N forms. Many studies had investigated competition for inorganic N between plants and microorganisms^12^,^13^,^14^. However, some studies^15^,^16^ also showed that plants could utilize organic N, such as free amino acids and peptides, found in the soil^17^,^18^. To understand plant-microbial interactions thoroughly, the acquisition of inorganic and organic N by plants and microorganisms must be investigated comparatively^19^,^20^.

A temporal scale was suggested to be essential for exploring plant-microbial interactions^5^, because of highly seasonal dynamics in biotic and abiotic factors. Soil resources (e.g., water and N) available to plants and microorganisms vary temporally^21^ resulting in noticeable compartmentalization in N uptake by microorganisms and plants with seasons^22^, hence driving phenological changes in plant species. Alpine plants acquire more N for growth during the early growing season, while microorganisms sequester N only in late growing season^23^. In arctic and alpine soils, net N mineralization tended to occur during the winter months, while net N immobilization tended to dominate during the summer^15^,^24^,^25^. Similar patterns were observed in montane heath communities in Scotland, where microbial N was greater in autumn, after plant senescence, than early in the growing season, when microorganisms were strongly N limited^26^.

Besides temporal variables, spatial factors also influence plant-microbial interactions significantly. For examples, root biomass^27^, microorganisms density^28^, and nutrients^29^,^30^ availability generally decrease as soil depth increases. A previous study investigated dynamics of NH_4_^+^ and NO_3_^−^ over a 2-year period in a deciduous woodland, showing the peaks of nutrient concentration only occurred in localized areas and was not predictable^30^. These studies confirmed the importance of spatio-temporal variations for understanding ecological processes. However, how plants and microorganisms acquire available N has not been well quantified over soil depth during the growth season.

Temperate grasslands are widely distributed across the Eurasian continent, known as “the Eurasian steppes”, connecting West and East Europe, Central Asia, China, South Asia, and the Middle East^31^. The Eurasian steppe is one of the largest temperate grasslands and play an important role in the global C and N cycles. Our previous study showed that spatio-temporal variations determine plant-microbial competition for inorganic N in alpine meadows^14^, but it remains unclear in temperate grasslands. To further emphasizing the importance of spatio-temporal influences on plant-microbial interactions crossing different habitat types, this study quantified N acquisition by plants and microorganisms over soil depth during the growing season using a short-term ^15^N labeling field experiment in Inner Mongolia. We aimed to test two hypotheses: (1) plants and microorganisms could partition different chemical N forms over spatial and temporal scales, and (2) plants more effectively acquire organic and inorganic N from the top soil than from the lower soil layers. Plants have co-evolved with microorganisms to form an extended root system helping them access soil area with higher N concentration, e.g. subsoil^32^. Comparing to plant roots, microorganisms are more attached to local soil particles and have little mobility to explore higher N locations. To coexist with strong N uptake system of plant roots, microorganisms either become stronger competitors or develop chemical and/or temporal niche differentiation to reduce competition intensity in the upper soil layer. Therefore, clarifying the aforementioned hypotheses will allow us to assess how plants and microorganisms acquire available N through chemical, spatial and temporal niche differentiation.

## Results

### Dynamics of plant and soil nitrogen pools

With season processing from July to September, the significance of different N pools and forms varied in different soil depths (Table 1). The plant N pool decreased by 35% over time, from 7,208 mg N m^−2^ in July to 4,715 mg N m^−2^ in September. There were no significant differences in the shoot N pool among three months, but the root N pool was higher in July than in both August and September. The microbial N pool was the largest in August, but its size in July and September relied on soil depth. Over soil depths, it was larger in the upper soil layer (0–5 cm) than the deeper soil layer (5–15 cm) in all three months (Table 1). Both NO_3_^−^ and NH_4_^+^ pools showed a similar spatio-temporal pattern. Their size was the largest in August but the smallest in July in the upper soil layer, while it increased from July to September in the deeper soil layer (Table 1). The glycine pool remained stable in the upper soil layer but showed maximum in the deeper layer in August. It was also the lowest pool, about 15% of the sum of all three N forms, comparing to the other two inorganic N pools in both soil depths (Table 1). Both spatial and temporal factors contributed to the dynamics of dominant NO_3_^−^ and NH_4_^+^ but only temporal factor influenced the size of organic N pool.

### Microbial nitrogen uptake

**Figure 1:**
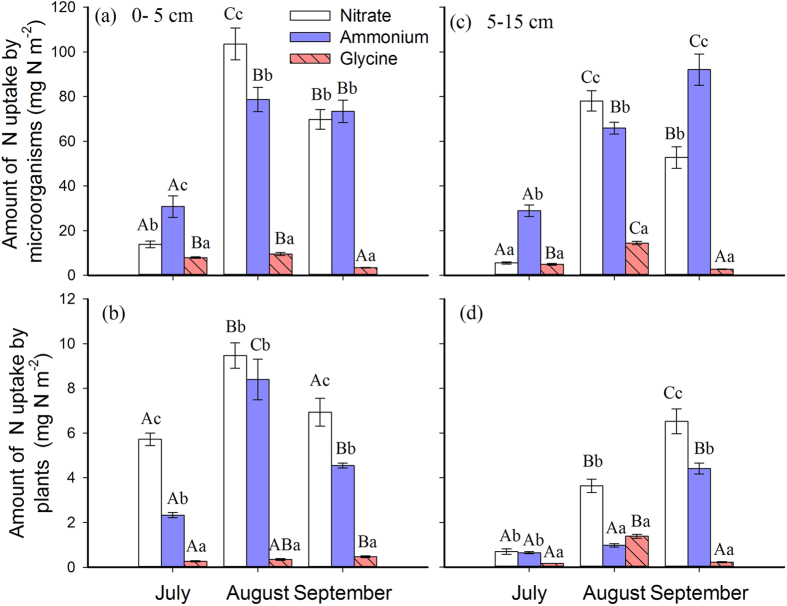
N uptake from NH_4_^+^, NO_3_^−^ and glycine by microorganisms and plants within 20 hours after ^15^N injection (10 mg ^15^N m^−2^) at soil depths (at 2.5 cm and 10 cm) during growing season (July, August and September). Values are presented as means ± 1SE (n = 4 replicates). Different small letters above each bar indicate significant difference of N uptake for nitrate, ammonium and glycine while different capital letters above each bar indicate significant difference of N uptake between seasons at P < 0.05.

Forms of N and temporal factor showed strong influence on microbial N uptake (Table S1 and Fig. S1). In the upper soil layer, the highest uptake of all three N forms occurred in August, while the lowest uptake of NH_4_^+^ and NO_3_^−^ in July and glycine in September (Fig. 1a). In the deeper soil layer, microbial uptake of NO_3_^−^ and glycine was significantly highest in August, but uptake of NH_4_^+^ significantly increased from July to September gradually (Fig. 1c).

**Figure 2:**
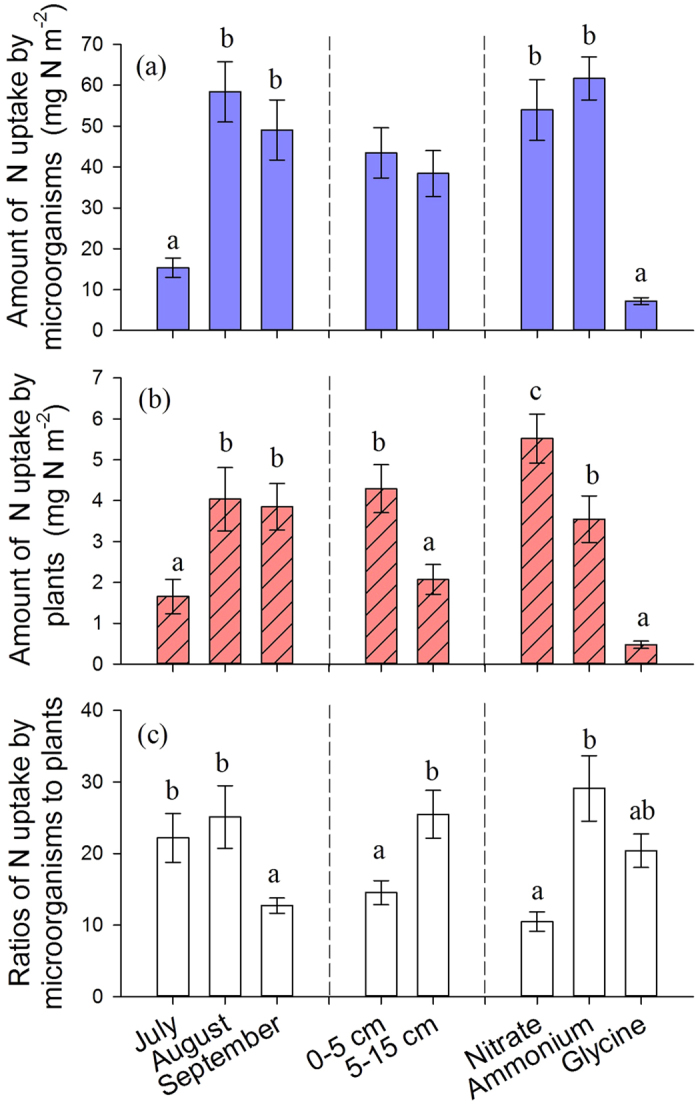
The main effect of month, soil depth and N chemical form on amount of N uptake by soil microorganisms, plants and ratios of N uptake by microorganisms to N uptake by plants in a temperate grassland. Bars and errors show means ± 1SE (n = 24 for effect of month and N form; n = 36 for effect of soil depth). Bars sharing the same letter are not different between treatments at *P* < 0.05.

The total N uptake by microorganisms was higher in August and September than in July, but similar between 0–5 cm and 5–15 cm soil layers (Fig. 2a). Compared to glycine, NO_3_^−^ and NH_4_^+^ were the preferable N forms for microorganisms.

### Plant nitrogen uptake

Forms of N, temporal, and spatial factors showed significant effects on plant N uptake (Table S1 and Fig. S1). Plants took up more NH_4_^+^ and NO_3_^−^ from the upper soil layer in August than in July and September (Fig. 1b). In the deeper soil layer, NH_4_^+^ and NO_3_^−^ uptake by plants significantly increased across the growing season, and glycine uptake by plants was the highest in August (Fig. 1d).

The total N uptake by plants was preferable from the upper soil, where was significantly higher uptake in August and September than in July. The most desirable form for plants was NO_3_^−^, followed by NH_4_^+^ and glycine (Fig. 2b and Fig. S1).

### Plant-microbial competition

**Figure 3:**
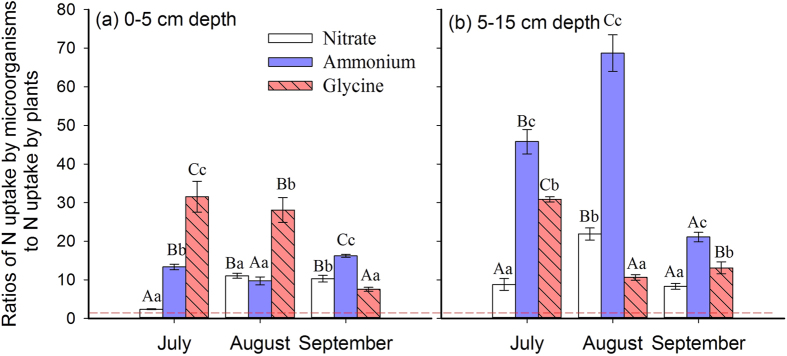
Ratios of N uptake by microorganisms to N uptake by plants from NH_4_^+^, NO_3_^−^ and glycine 20 hours after ^15^N injection at different soil depths during the growing season (July, August and September) in a temperate grassland. The dashed line below corresponds to 1.0 (identical N uptake by microorganisms and plants). The values are presented with the means ± 1SE (n = 4 replicates). Different small letters above each bar indicate significant difference of N uptake for nitrate, ammonium and glycine while different capital letters above each bar indicate significant difference of N uptake between seasons at P < 0.05.

Multifactorial ANOVA showed that forms of N, spatial and temporal factors, and their interactions had significant effects on the ratios of N uptake by microorganisms to N uptake by plants (N_MB_:N_PL_) (Table S1). Overall, the N_MB_:N_PL_ uptake ratios ranged from about 2.4 to 68.7 (Fig. 3), indicating microorganisms were the superior competitor over plants for available N in short term.

In the upper soil layer, N_MB_:N_PL_ ratios for glycine decreased, but increased for NO_3_^−^ from July to August. In the deeper soil layer, N_MB_:N_PL_ ratios for NH_4_^+^ and NO_3_^−^ were the highest, and the ratios for glycine was the lowest in August (Fig. 3). If evaluating the factors (i.e., forms of N, spatial and temporal factors) independently, N_MB_:N_PL_ ratios were higher in the deeper soil layer, in July and August and for NH_4_^+^ (Fig. 2c).

### Chemical, spatial and temporal partitioning of N between plants and microorganisms

**Figure 4:**
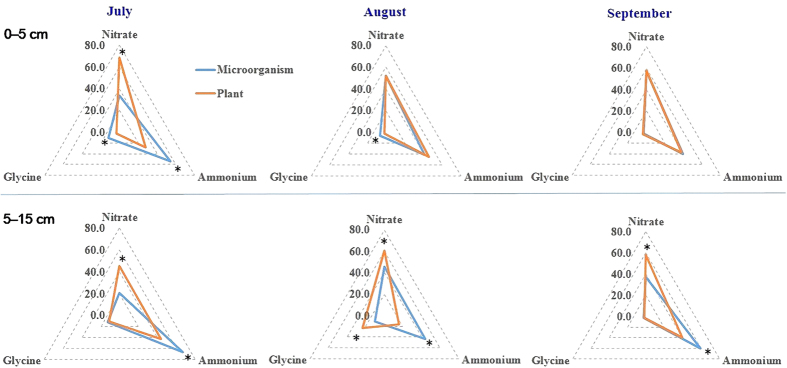
Chemical niche shifts for plants and microorganisms over soil depths during the growth season in a temperate grassland. The axes of x, y and z represent the contribution of ammonium, nitrate and glycine to total N uptake (as %). Asterisks indicate significant difference (P < 0.05) between plants and microorganisms.

Chemical niche differentiation for plants and microorganisms was demonstrated by spatial and temporal variables in the observed temperate grassland. In July, plants favored NO_3_^−^ while microorganisms preferred NH_4_^+^ in the upper soil layer (Fig. 4 and Fig. S1). Although the separation was not always so clear in August and September, both plants and microorganisms acquired similar proportion of all three N forms, showing overlap chemical niche. In the deeper soil layer, plants and soil microorganisms demonstrated distinct chemical niches over the whole vegetation period, with plants preferring NO_3_^−^ and microorganisms preferring NH_4_^+^ (Fig. 4).

## Discussion

Nitrogen acquisition by plants and microorganisms from NH_4_^+^, NO_3_^−^, and glycine was evaluated by conducting short-term ^15^N tracer experiments in a temperate grassland in Inner Mongolia. The spatio-temporal competition between plants and microorganisms was investigated in two soil layers throughout three months during one growing season.

### Microbial nitrogen uptake

Numerous studies found that heterotrophic microorganisms preferred NH_4_^+^ more than NO_3_^−9,^ 33 due to the energy costs for NO_3_^−^ reduction^34^, while some demonstrated that microorganisms also taken up more NO_3_^−^ than NH_4_^+^ in a pot experiment^35^. Our results showed that NO_3_^−^ was the main uptake in August indicating microbial preference of N forms varied throughout growing season (Fig. 1). A shift of microbial community composition during the growing season^36^ could change in the N preference, but further investigation in microbial community dynamics should be conducted to clarify this point.

It has been suggested that microorganisms take up glycine effectively in unimproved grasslands but not in improved grasslands^19^. Glycine provides both C and N for soil microorganisms that are often limited by available C and energy^37^,^38^, but its uptake was the lowest in this study. This could be due to low concentrations of glycine, which were 4–10 folds less than the concentrations of NO_3_^−^ or NH_4_^+^, at the study site (Table 1). Although glycine concentration did not change throughout the growth season, its contribution to microbial N uptake decreased. Because microbial organic N uptake is down regulated by carbon availability^39^, we speculated that microorganisms in the Ah horizon, with high C input via rhizodeposition^40^ and litter input at late growing season of temperate grasslands were not C limited. Therefore, reduced microbial glycine uptake was probably due to higher C availability at late stages of the growing season. Another explanation is that living root and its symbiotic fungi could become less active, which would result in lower plant acquisition of organic N^41^.

Temporal scale is an important factor when trying to understand ecological processes^4^. Here, we showed that growing season had strong effect on microbial N uptake, e.g., the lower uptake in July than in the rest of season (Fig. 2a). This pattern differs from previous observations in alpine grasslands, where microorganisms acquired more N at the end of the growing season^23^. In this study, microbial N uptake was related to plant performance and soil N availability, e.g., in August, the increasing microbial biomass resulted in higher microbial N uptake, while in July, higher plant biomass led to lower microbial N uptake (Table 1, Fig. 1). Based on our results, we found that microbial N uptake was positively correlated with increases in available N, microbial biomass N pool and plant N uptake, but negatively correlated with increased plant biomass and ratios of plant N uptake to microbial N uptake. This indicated that microbial N uptake could be facilitated by plant N uptake but reduced by plant competition. Possible explanation was that strong N uptake by plants might increase water flow which delivered available N to the root surface, where more accessible to the microorganisms in the rhizosphere. Microbial N uptake decreased with increased ratios of (N_PL_:N_MB_) plant N uptake to microbial N uptake, indicating that stronger plant competition reduced microbial N uptake.

Microbial biomass generally decreases with soil depth^28^. Subsequently, microbial N uptake is expected to decrease with increasing depth. We found that microbial N uptake from the surface (0–5 cm) was nearly equal to that from the 5–15 cm layer (Fig. 2a). The microbial biomass density was twice higher in the upper soil layer than the 5–15 cm layer. This suggested that the upper soil played a more important role in nutrient cycling in temperate grasslands, because it contains higher concentration of N source and microbial density in a smaller volume. Overall, microbial N uptake demonstrates temporal differentiation with preference for N form, but no spatial differentiation.

### Plant nitrogen uptake

Plant N uptake was strongly affected by N form and soil depth as well as interactions between these factors (Fig. S2). At the study site, regardless the low soil moisture and higher NH_4_^+^ concentration than NO_3_^−^ in the soil (Table 1), plants strongly preferred taking up NO_3_^−^ than NH_4_^+^ (Fig. 2b). The distinct chemical properties of NO_3_^−^ and NH_4_^+^ might be the probable cause: NO_3_^−^ is more mobile in soil solution and readily for plant to absorb while the positive charge of NH_4_^+^ restrained its mobility by organo-mineral complexes^42^. Low soil moisture together with high soil organic matter content decreases the rate of NH_4_^+^ delivery to the root surface^43^,^44^,^45^. Another reason could be that an increased concentration of cations such as K^+^, Ca^2+^, and Mg^2+^ at the studied site steered the N form preference of plants to NO_3_^−^. Strong preferential uptake by plants led to the decrease of NO_3_^−^ concentration.

Although numerous studies using ^13^C and ^15^N dual amino acids confirmed that temperate grasses can use organic N in intact form of organic N^19^,^20^, we showed that uptake of glycine is very low compared to inorganic N forms. We could not quantify the contribution of intact glycine uptake because we only used ^15^N labeled glycine. Because the rate of glycine mineralization would theoretically be similar to ammonium which was not observed here (Fig. 1), without dual labeling method, our results still provided judicious estimates of maximal uptake of glycine molecules by plants. We concluded that glycine is not important for plants and microorganisms in the observed temperate grassland.

Plants took up more N in August and September when their biomass was lower, which could be explained by the distinct N requirement at different growth stages. More N was acquired during reproductive stages, August and September, than regular growing stage, July, by the plants. Although the concentrations of available N were similar between the upper soil and the 5–15 cm soil layer, plants still acquired more than 67% of their total N from the upper soil (Fig. 2). Spatial preference pattern was consistent with the root distribution, e.g. root N pools show more than 80% of roots in the upper soil (Table 1). This supports our hypothesis that plants effectively acquire organic and inorganic N from the top soil in the temperate grassland. In brief, plant N uptake showed temporal, spatial differentiation, as well as different N form preferences.

### Plant-microbial competition

A spatio-temporal context is a prerequisite for the exploration of plant-microbial competition. This competition is regulated by the differences in N availability, microbial distribution, as well as temporal differences in microbial and root turnover^9^. Our previous study had demonstrated that spatio-temporal variations corresponding to root biomass controlled plant-microbial competition for inorganic N in alpine grasslands^14^, but our results in temperate grassland did not support this hypothesis. This indicates that alpine and temperate grasslands have distinct plant-microbial competition patterns. A possible explanation is that N limitation in temperate grasslands may not be as severe as in alpine ecosystems^43^. Another explanation is that the typical herbaceous plants have relatively larger root and/or rhizome systems in alpine habitats than in temperate environments, leading to higher competition from plants in alpine grasslands. Microorganisms outcompete plants for N in short-term as indicated by ratios of N_MB_:N_PL_ higher than one. These ratios can be even underestimated due to the limitations of the chloroform fumigation-extraction approach. The ratios of N_MB_:N_PL_ in all cases ranged from 2 to 69 in different months, N forms, and soil depths. Except for NO_3_^−^ in July, microorganisms took up at least seven times more N from all three N forms than plants did (Fig. 3). These values were higher than those observed in annual grasslands^12^, indicating that microorganisms strongly outcompeted plants for available N in temperate grasslands, as previously suggested over short-term periods^1^,^9^.

Although microorganisms were more effective competitors for available N than plants, both players demonstrated a clear chemical niche differentiation over depths and months (Fig. 4) confirming our hypothesis that plants and microorganisms could partition different chemical N forms over spatial and temporal scales to reduce competition. In the 0–5 cm soil layer in July, the preferences for NH_4_^+^ by microorganisms and for NO_3_^−^ by plants reflect chemical niche differentiation (Fig. 4) but intensified competition for N in August and September overlapped chemical niches (Fig. 4). In comparison, plants and microorganisms demonstrated a clear chemical niche differentiation in the deeper layer cross months^10^. Microorganisms acquired more than 54% and plants acquired over 75% of their total N from the upper soil in July and August, while both took up similar amounts from both soil layers in September (Fig. S1). This reflects no general spatial niche differentiation between microorganisms and plants (Fig. 4).

In summary, the maximal N pool in plants was in July, while microbial biomass N pool was the highest in August. The maximal inorganic N (NO_3_^−^ + NH_4_^+^) pool and glycine-N pool fluctuated with months and soil depths. The uptake of available N by microorganisms was higher in August and September than in July. Soil depth had no significant effect on microbial uptake, while inorganic forms were preferable. This suggests that microbial N acquisition shows temporal differentiation with preference for different forms of N, but no spatial differentiation. The most preferable form of N was NO_3_^−^ regardless soil depths and months but overall available N uptake was from upper 5 cm soil layer. Across months, plants took up less N in July than in August and September. This reflects that plants demonstrate chemical, temporal and spatial differentiation for N uptake. The N_MB_:N_PL_ uptake ratios ranged from about 2.4 to 68.7, indicating that microorganisms strongly outcompeted plants for inorganic and organic N over soil depths and months. Although our study has some uncertainties by potential rapid N turnover and dynamics, our results showed that plants and microorganisms demonstrate chemical niche partition over soil depths and months. Such chemical niche partition can help plants and microorganisms to relieve the competition for N especially in deeper soil layer, where plants take minority of its N. In upper soil layer, where plant roots are very dense, taking up the majority of required N, neither chemical nor temporal niche separation is realized between plants and microorganisms. This upper layer is a place of strong competition for N between plants and microorganisms, playing an important role in N dynamics in the temperate grassland.

## Materials and Methods

### Study Site

The experiment was conducted at the Hulunbeier Prairie Ecosystem Station of the Chinese Agricultural Academy of Sciences, typical temperate steppe zone in Inner Mongolia Autonomous Region (49°21′8′′–49°22′4′′N, 120°2′14′′–120°7′25′′E, 620–630 m above sea level). Average annual temperature and precipitation during the past 20 years were 3.6 °C and 350 mm. Average temperature during the growing season, from early May to early October, was 15.8 °C, and most of the precipitation was concentrated in the summer, from May to August^46^. The rainfall and temperature data during the observed period were presented in Fig. S3. The dominant plant species are *Leymus chinensis* (Trin.) Tzvel., *Festuca ovina* Linn., *Artemisia tanacetifolia* Linn., *Pulsatilla turczaninovii* Krylov et Serg., *Artemisia dracunculus* Linn., and *Koeleria cristata* (Linn.) Pers. The soil is classified as chestnut soil, also known as Haplic Kastanozem^47^.

### Experimental layout

The field experiments were done in an area (50 m × 50 m) chosen for its uniformity in plant species diversity. To compare with our previous studies in alpine grasslands^11^, in this study we focused on the middle and late stages of the growing season. Thirty-two plots (15 cm × 15 cm) were set up in July, August, and September 2010, respectively. These plots were randomly divided into two (soil depth) groups (0–5 cm and 5–15 cm) based on previous observations on root distribution, with 16 plots for each group. Each soil depth group was randomly assigned to four different N form treatments, i.e., K^15^NO_3_ (99.19 atom% ^15^N enrichment), (^15^NH_4_)_2_SO_4_ (99.14 atom% ^15^N enrichment), glycine (99.04 atom% ^15^N enrichment) labeled with ^15^N, and control. These N forms were used, mainly considering that inorganic N (i.e., NH_4_^+^ + NO_3_^−^) and free amino acids are the important N sources for plants and soil microorganisms. Common to many studies^19^,^48^, glycine was used to measure amino-acid N uptake since it is one of the most abundant amino acids observed in soil solution of grasslands^49^. All solutions were a mixture of NH_4_^+^, NO_3_^−^ and glycine (1:1:1 N-NH_4_^+^/N-NO_3_^−^/N-glycine, 12.5 mg N L^−1^ for each N form), but only one form of tracer was ^15^N labeled at each treatment. The control treatment was only injected with H_2_O. Each treatment had four replicates. Labeled ^15^N tracers were injected at 2.5 cm depth for the upper soil layer (0–5 cm) group and at 10 cm for the deeper layer (5–15 cm) group. To make added ^15^N substrates completely mixed with existing soil pools, each plot was divided into 9 subplots (5 cm × 5 cm) and injected with 2 mL of the corresponding ^15^N solution at the center of the subplot based on the previous solution diffusion tests. The added total N amount was 30 mg N m^−2^ in each plot to avoid fertilization effect. There are major variables in this experiment: form of N resources (^15^N in NO_3_^−^, NH_4_^+^ or glycine); temporal (July, August or September), and spatial, the soil depth of ^15^N injection (2.5 or 10 cm).

### Sampling and analyses

Considering fast N transformation and the convenience for sampling, we collected samples 20 hours after the ^15^N tracer injection. All aboveground plant parts within each plot were clipped close to the soil surface with scissors. After clipping, four soil cores (5 cm in diameter) were randomly collected from 0–5 cm and 5–15 cm layers using a soil auger within the same plot to avoid possible shortcomings caused by inhomogeneous mixing. Soil samples were immediately brought to the laboratory at the field station, and were sieved through a 2-mm mesh. The sieved soil samples were stored at −20 °C until the microbial biomass N was measured. Total N and organic carbon in soil was measured on an elemental analyzer (EA 1112, CE Instruments, Milan, Italy) after carbonates were removed with acid addition. A pH analyzer was used to measure the supernatant with dry soil-water ratio of 1:2. Living roots were carefully picked up from the soils. These roots were rinsed with tap water, submerged in 0.5 mmol L^−1^ CaCl_2_ solution for 30 min, and then washed with distilled water to remove ^15^N from the surface of the roots. Aboveground parts and roots were dried at 80 °C for 48 h and weighed to measure dry mass. Dried plant roots and shoots were ground to a fine powder using a ball mill (MM2, Fa. Retsch, Haan, Germany). Aliquots (2 mg) of plant materials were weighed into tin capsules to analyze the total N and ^15^N:^14^N ratios using continuous-flow gas isotope ratio mass spectrometry (MAT253, Finnigan MAT, Bremen, Germany), coupled with ConFlo III device (Finnigan MAT, Bremen, Germany) and an elemental analyzer (EA 1112, CE Instruments, Milan, Italy). The frozen soils were used to measure microbial biomass N and its ^15^N content by chloroform fumigation-extraction procedure^25^. After the soils were left to defrost slowly, fifteen grams of soils were fumigated with chloroform for 24 h, then immediately extracted with 60 mL 0.05 mol L^−1^ K_2_SO_4_. An additional soil sample was extracted without fumigation. The K_2_SO_4_ extracts were immediately frozen and freeze-dried for analysis of N content and the ^15^N:^14^N ratios using continuous-flow gas isotope ratio mass spectrometry (MAT253, Finnigan MAT, Bremen, Germany). The soils from the control treatments were extracted with 0.05 mol L^−1^ K_2_SO_4_ and the extracted were used to measure NO_3_^−^-N and NH_4_^+^ -N by an auto-analyzer (AA3, Bran-Luebbe, Germany). Soil glycine concentrations were measured by high-performance liquid chromatography (Waters 515, Waters Inc., USA) from the same extracts^50^. It can underestimate microbial biomass N and ^15^ N by using chloroform fumigation-extraction procedure on defrosted soils, because the extraction from control soils (extracted without fumigation) will for sure contain N and ^15^ N released from the microbial cells damaged by freezing-defrosting. This amount cannot be estimated without testing-comparison of chloroform fumigation-extraction made on frozen and fresh soils.

### Calculations and statistics

The calculations followed most ^15^ N labeling studies to examine plant-microbial competition for N^1^. A major assumption, based on previous studies which showed mean residence times of soil NH_4_^+^ about 2.8 ± 0.5 d and comparable ammonization and nitrification rates, is that the N-forms does not change during the 20-hour period of labeling in temperate grasslands in Inner Mongolia^51^,^52^. Additional assumptions are that there is no change in the soil N pool during the labeling period and no abiotic ammonium fixation in the soil. If abiotic ammonium fixation happened, it would lead to simultaneous overestimation on N acquisition by plants and microorganisms but would not overestimate their competition. The ^15^ N atom% excess (APE) was calculated as the percentage difference between the ^15^ N treated samples and the control. Uptake of ^15^N by plants (mg ^15^N m^−2^) was calculated by multiplying biomass (g m^−2^), APE, and N content (mg N g^−1^ DW). Microbial ^15^N uptake (mg ^15^N m^−2^) was calculated as the difference in the mass of ^15^N between fumigated and non-fumigated soil samples. Actual N uptake from soil by plants or microorganisms was calculated by multiplying the uptake of ^15^N by the corresponding N pool (i.e., NO_3_^−^, NH_4_^+^, or glycine) in the soil, divided by the total amount of ^15^N added^10^,^11^ as following: U_N_ = U_15N_ (M_N_/M_15N_), where M_15N_ is the total mass (g m^−2^) of ^15^N–labelled N injected per plot; M_N_ is the mass of available N species (i.e., NO_3_^−^, NH_4_^+^, or glycine) measured in soil; U_15N_ is uptake (g m^−2^) of ^15^N from the source M_15N_; and U_N_ is uptake of available N from the source M_N_. A recovery coefficient was not applied in those studies due to the uncertainties caused by temporal variations in the extractability of N and the variability in incorporation efficiency into the cytoplasmic (soluble) vs. structural (insoluble) components^53^. Within the first 20 hours, ^15^N would be not incorporated into structural compounds, but remains mainly in cytoplasm. Therefore, we did not use K_EN._ The results presented here represent a conservative estimate of the microbial biomass pool and isotope content. Competition between plants and soil microorganisms for N, was measured as the ratio of N uptake by microorganisms to N uptake by plants (N_MB_:N_PL_). The contribution of different soil layers (0–5 cm and 5–15 cm) was estimated by dividing the N uptake for an individual layer by the total N uptake from both layers. The contribution of different months (July, August, and September) was estimated by dividing N uptake for an individual month by the total N uptake from all three months. The contribution of different N forms (NO_3_^−^, NH_4_^+^, and glycine) was estimated by dividing N uptake of an individual N form by the total N uptake for all three N forms.

Means values and their associated errors were presented in figures and tables. Multifactorial analysis of variance (ANOVA) was used to estimate the effects of N form, month, soil depth, and their interactions on N uptake by microorganisms (N_MB_), N uptake by plants (N_PL_), and their ratio (N_MB_:N_PL_), by using the SPSS16.0 software package (SPSS Inc., Chicago, IL, USA). The contributions of the factors and their interactions to the total variance were calculated by dividing the respective type III sum of squares by the total sum of type III sum of squares from the multifactorial ANOVA. All differences were tested at P < 0.05.

## Additional Information

**How to cite this article**: Liu, Q. *et al.* Nitrogen acquisition by plants and microorganisms in a temperate grassland. *Sci. Rep.* **6**, 22642; doi: 10.1038/srep22642 (2016).

## Supplementary Material

Supplementary Information

## Figures and Tables

**Table 1:** Nitrogen in organic and inorganic pools, microbial biomass and plants in a temperate grassland in July, August and September of 2010.

		July	August	September
Nitrogen pools		mg N m^−2^		
Plants	Total biomass	7208.3 ± 264.1B	4755.5 ± 201.08 A	4715.9 ± 171.5 A
	Aboveground	3445.0 ± 201.2	2960.1 ± 189.4	2951.0 ± 183.3
	Roots (0–5 cm)	3056.1 ± 169.0Bb	1547.1 ± 95.3Ab	1476.9 ± 65.8Ab
	Roots (5–15 cm)	707.2 ± 58.9Ba	248.3 ± 15.1Aa	300.5 ± 22.2Aa
Soil				
0–5 cm				
	NO_3_^−^	144.8 ± 10.7Ab	301.7 ± 43.3B	285.7 ± 36. 8Ba
	NH_4_^+^	188.2 ± 20.1b	308.2 ± 36.2	249.0 ± 45.1a
	Glycine	33.1 ± 2.2	30.9 ± 3.2a	35.1 ± 0.4
	Microbial biomass	2534.2 ± 200.3 A	4565.1 ± 173.1Cb	3256.2 ± 254.0Bb
5–15 cm				
	NO_3_^−^	48.0 ± 2.0Aa	287. 8 ± 42.3B	353.6 ± 32.1Bb
	NH_4_^+^	114.7 ± 22.4Aa	312.1 ± 36.9B	404.6 ± 32.1Bb
	Glycine	29.4 ± 2.7 A	77.3 ± 4.3Bb	35.3 ± 0.6 A
	Microbial biomass	2403.2 ± 238.7 A	3794.8 ± 206.6Ba	2386.4 ± 277.7Aa

The values for various N pools are presented as means ± 1 SE of 8 replicates. Capital letters indicate significant difference of N pools between seasons at P < 0.05 levels and small letters indicate significant difference of N pools from soil depths at P < 0.05 levels.
