# Supplementary material for: Nitrogen acquisition by plants and microorganisms in a temperate grassland
Source: Sci Rep. 2016 Mar 10;6:22642. doi: 10.1038/srep22642 (PMC4785369; doi:10.1038/srep22642)
Supplement: Supplementary Information [file srep22642-s1.pdf]

Nitrogen acquisition by plants and microorganisms in a temperate grassland

Qianyuan Liu, Na Qiao, Xingliang Xu, Xiaoping Xin, Jessie YcHan, Yuqiang Tian, Hua Ouyang, YakovKuzyakov

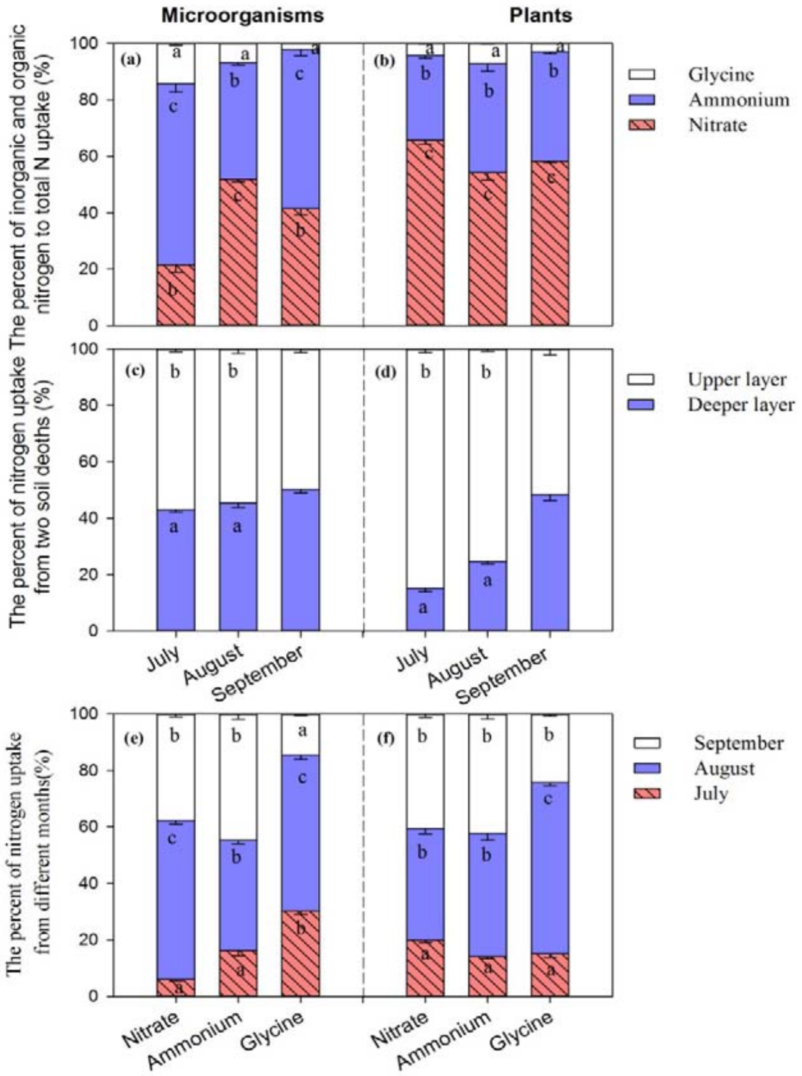

Figure S1. The percentage of NH_4^+-N, NO_3^--N and glycine-N to total nitrogen uptake by microorganisms (a) and plants (b), the percentage of nitrogen uptake by microorganisms (c) and plants (d) from the upper soil (0–5 cm) and the second soil layer (5–15 cm), and the percentage of nitrogen uptake by microorganisms (e) and plants (f) in July, August and September in a temperate grassland. Values are presented with the mean ± 1SE (n=4 replicates). Bars sharing the same letter are not different between treatments at *P*<0.05.

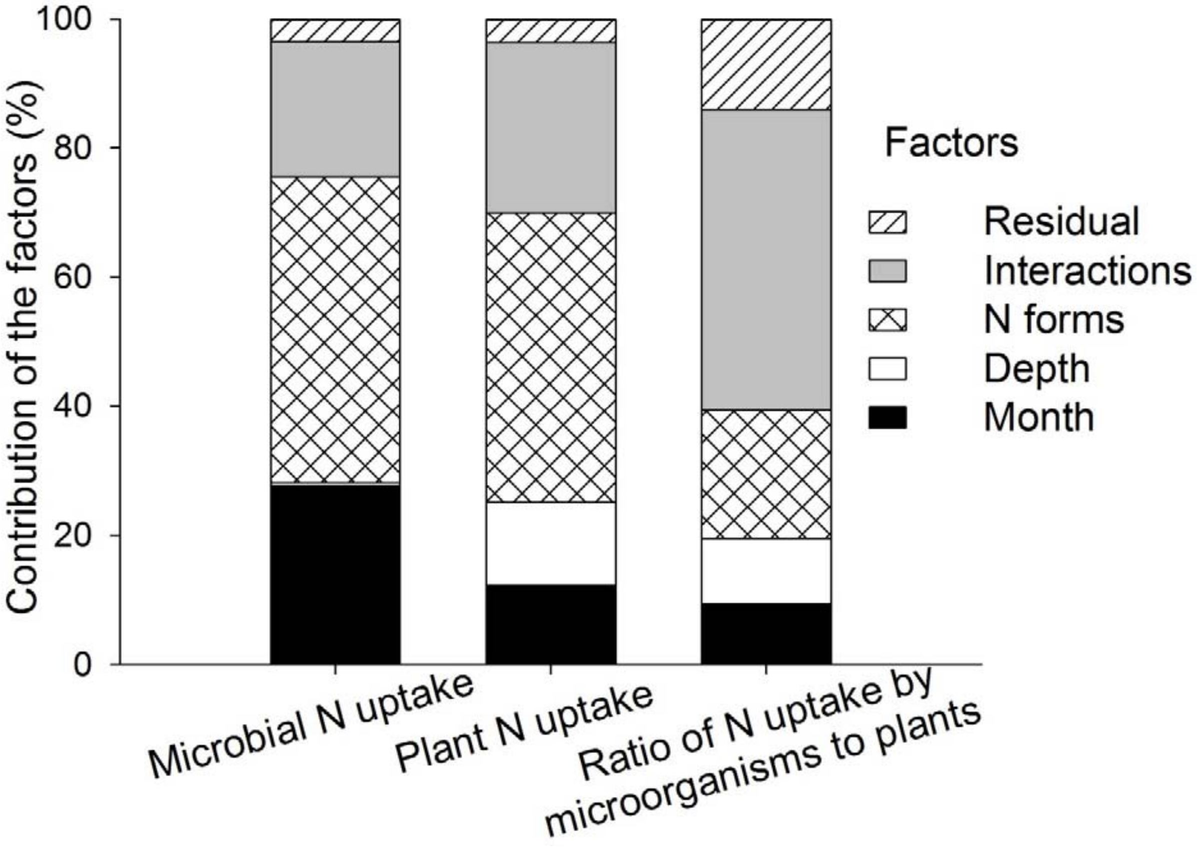

Figure S2. Contributions of the 3 factors: month, soil depth, N form and their interactions to thevariance in microbial N uptake, plant N uptake and their ratios. Interactions show the sum ofinteractions among all the factors. Residuals depict the variance unexplained by either factors orfactor interactions.

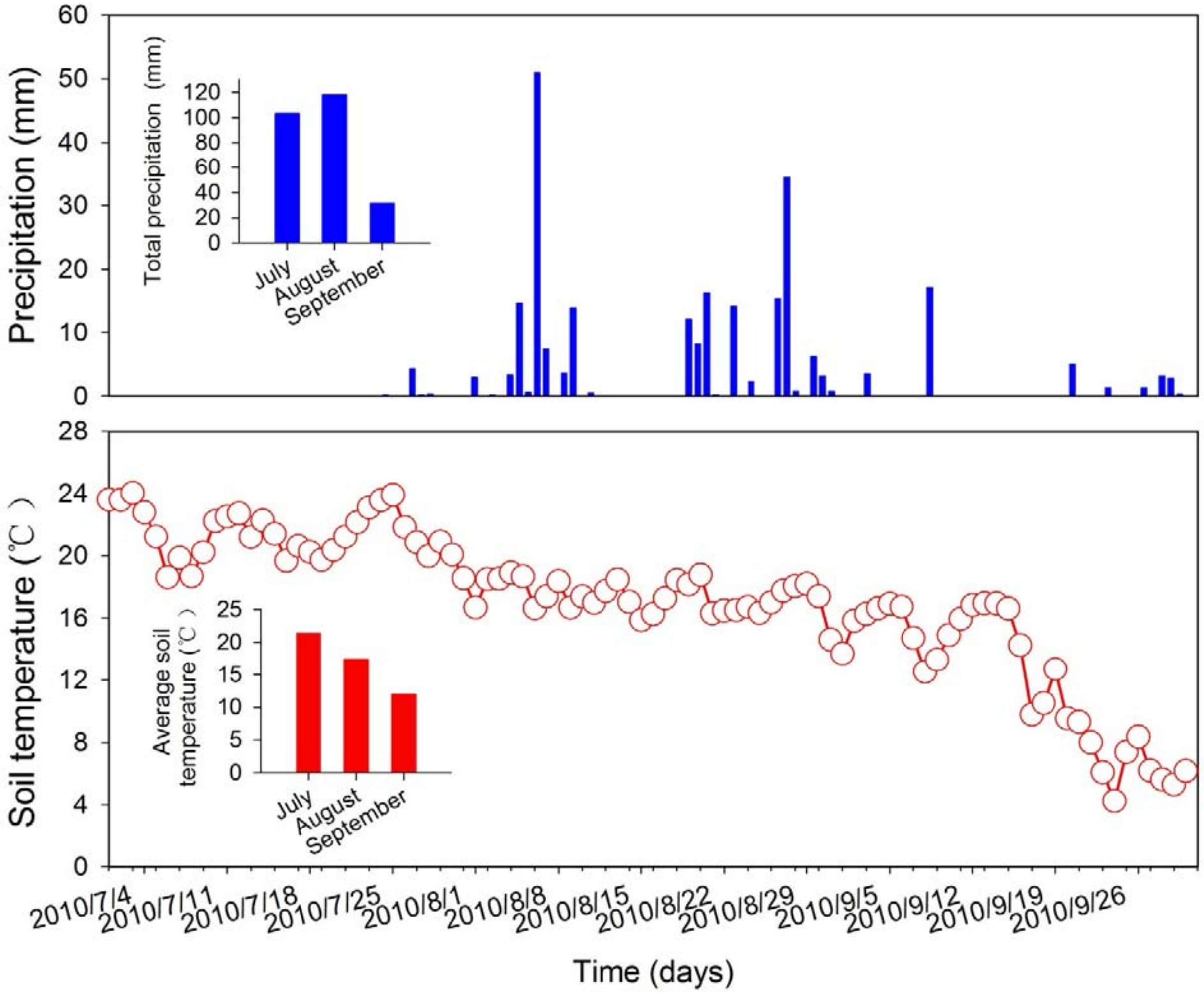

Figure S3. The precipitation (mm) and temperature (°C)during the experimental period in temperate steppe of Inner Mongolia in northern China.

Table S1. Multifactorial analysis of variance for the effectson N form, month, soil injection depth and their interaction on N uptake by microorganisms and plants in a temperate grassland. The competition for N between microorganisms and plants is presented as ratio of N uptake by microorganisms (N_{MB}) to N uptake by plants (N_{PL}).The P values for significant effects and interactions are in bold.

	Microbial N uptake		N uptake by plants		N_{MB}:N_{PL}	
Source of variation	F values	Pvalues	F values	Pvalues	F values	Pvalues
Month	211.1	**<0.001**	90.8	**<0.001**	36.0	**<0.001**
Depth	7.0	0.010	193.5	**<0.001**	79.2	**<0.001**
N form	361.7	**<0.001**	333.9	**<0.001**	76.9	**<0.001**
Month × Depth	3.9	0.026	47.6	**<0.001**	11.7	**<0.001**
Month × N form	66.7	**<0.001**	16.1	**<0.001**	17.2	**<0.001**
Depth × N form	11.5	**<0.001**	59.9	**<0.001**	80.2	**<0.001**
Month × Depth × N form	4.9	0.002	28.1	**<0.001**	27.2	**<0.001**

Table S2. Characteristics of the top 10 cm (Ah) of soil in a *Leymuschinensis* steppe in Inner Mongolia of northern China. Means± 1SE of 12 replicates are presented.

Soil properties	Temperate steppe
pH(H_2O)	6.7±0.1
C:N ratio	9.6±0.1
Organic C content (%)	3.56±0.08
Total soil N (%)	0.37±0.01
